# Supplementary material for: Polyvinyl chloride-based dielectric elastomer with high permittivity and low viscoelasticity for actuation and sensing
Source: Nat Commun. 2023 Mar 17;14:1483. doi: 10.1038/s41467-023-37178-5 (PMC10023783; doi:10.1038/s41467-023-37178-5)
Supplement: Supplementary file 1 — Supplementary Information [file 41467_2023_37178_MOESM1_ESM.pdf]

Supplementary Information

for

Polyvinyl chloride-based dielectric elastomer with high permittivity and low viscoelasticity for actuation and sensing

by Huang, et al

Supplementary Notes

Supplementary Note 1: Effect of DOP on Mechanical Properties and Viscoelasticity of PVCg

To decrease the stiffness of PVC, the plasticizer of DOP was introduced into PVC. Seen from **Supplementary Fig. 1**, the pure PVC has a very high modulus of 23.2 MPa (**Supplementary Fig. 1a**), which is not suitable for actuation and sensing applications. After introduction of DOP, the elastic modulus of the PVCg decreased with increasing DOP concentration. The counted elastic moduli were 0.54 MPa for the mass ratio of 1:1 (PVC: DOP), 0.15 MPa for 1:2, 0.04 MPa for 1:3, 0.02 MPa for 1:4 (**Supplementary Fig. 1b, 1c**).

Supplementary Fig. 2 compared the dynamic viscoelasticity of PVCg elastomers with varying DOP concentrations. The PVCg elastomer with the ratio of 1:1 presented the highest storage modulus (G') (**Supplementary Fig. 2a**), and the lowest loss modulus (G'') (**Supplementary Fig. 2b**), thus this elastomer exhibited the lowest mechanical loss ($\tan \delta = G''/G'$) (**Supplementary Fig. 2c**). With increasing DOP concentration, the viscoelasticity showed an evidently increased trend. Under constant load of 60 g for 6 hours, the creep-induced elongation increased from 25 % of the mass ratio of 1:1 to 60 % of 1:4 ratio (**Supplementary Fig. 3a-3d**). Similarly, under constant strain of 100 % for 10 min, the recorded stress attenuation increased from 11.41 % of 1:1 ratio to 33.99 % of 1:4 ratio（**Supplementary Fig. 3e-3f**).

Supplementary Fig. 4a plotted the breakdown strength as a function of DOP concentration. The self-casting PVC plastics presented a breakdown strength of 41.19 V/μm, closing to the commercial PVC plastics of around 45 V/μm [1]. Introduction of DOP evidently weakened

PVC's breakdown strength, the detected breakdown strengths of PVCg decreased from 41.19 V/μm to 12.60 V/μm with increasing DOP concentration.

Supplementary Note 2: Synthesis and Characterization of Cyanoethyl Cellulose (CEC)

CEC was prepared from microcrystalline cellulose (MC) *via* the Michael addition reaction [2] as shown in **Supplementary Fig. 5a**. Total synthesis process was monitored by transmittance FTIR (Bruker IFS66/S, Germany). For MC, a broad absorption peak appeared at 3000-3650 cm^{-1}, which was attributed to the stretching vibration of the O-H bond (**Supplementary Fig. 5b**). Curve of CEC presented a strong, sharp absorption peak at 2250 cm^{-1}, which belonged to the C≡N bond from acrylonitrile.

Degree of substitution (DS) was counted by the **Supplementary Equation 3**. Elements of H, C, and N of MC and CEC were measured by an Elemental analyzer (VARIO ELIII, Germany). The element contents of H, C and N were 6.24, 43.41, 0 % for MC and 5.96, 54.82, 12.48 % for CEC, respectively (**Supplementary Fig. 5c**). The DS of CEC was calculated from the content of N element, its mean value was 2.74 after 3 times detections.

The crystalline structures of MC and CEC were monitored by XRD (Bruker D8 ADVANCE, Germany) analysis. MC presented a typical cellulose Type I structure [3] with a high crystallinity (*DC* = 77.60 %) (**Supplementary Equation 4, Supplementary Fig. 5d**). After cyanoethylation, the diffraction peaks of Type I structure disappeared, and a new diffraction peak appeared at 10.4° [4]. Its intensity lowered and appeared at a smaller angle, and the counted crystallinity was 27.75 %.

A thermal analyzer (TGA, SDTQ600) was used to compare the thermal stability of MC and

CEC. The initial decomposition temperatures of MC and CEC were 243 and 165 °C under N_2 atmosphere, respectively (**Supplementary Fig. 5e**).

Dielectric properties of dielectric permittivity and AC conductivity of MC and CEC were tested by using an impedance analyzer (Agilent 4294A, USA) with loading frequencies of 40 to 10^7 Hz. Powder of MC or CEC was pressed into disc sample by a tablet press (769YP-24B, China). The diameter was set to 10 mm, and the thickness was set ~1.0 mm. The steady permittivity @ 1.0 k Hz of the pure MC was 9.98 (**Supplementary Fig. 5f**). After introduction of cyanoethyl group, due to the dipolar polarization [5] of the cyanoethyl group, the steady permittivity @ 1.0 k Hz of the pure CEC increased to 12.32 (**Supplementary Fig. 5f**).

Supplementary Note 3: Discussion on Transmittance, Mechanical Properties, Viscoelasticity and Dielectric Properties of PVCg and CEC/PVCg Elastomers

Optical transmittances of PVCg and CEC/PVCg elastomers were tested by using a UV/visible spectrometer (Shimadzu UV-3600i PLUS, Japan). In the wavelength range of 400~1000 nm, their minimum transmittances were 89.54 and 85.53 %, respectively (**Supplementary Fig. 6**).

Tensile tests were performed by using an automated materials testing system (UTM2202, Shenzhen) at room temperature with a crosshead speed of 10 mm/min (**Supplementary Fig. 7**). Each elastomer with dimensions of 5.0 × 0.4 cm^2 (length × width) was clamped by a pair of pneumatic grips. Data of the tensile strength, Young's modulus, and the elongation ratio (at break) were obtained from the stress–strain curve, and shown in **Supplementary Table 1**. Results showed that, Young's modulus (Y) evolved from 0.15 to 0.51 MPa with CEC loadings

increasing from 0 to 17 %, while the elongation at break decreased to 76 % as compared to the PVCg elastomer.

Dielectric constants, dielectric losses and AC conductivities of CEC/PVCg elastomer were tested by using the former impedance analyzer (Agilent 4294A, USA) (**Supplementary Fig. 8**). Each CEC/PVCg elastomer was cut into a same disk-shaped specimen with a diameter of 10 mm and a thickness of ~1.0 mm. In **Supplementary Fig. 8a**, because the electronic (or dipolar) polarization always lags behind the change of electrical field [6], each CEC/PVCg elastomer presented a decreasing dielectric permittivity and dielectric loss with frequency. With CEC loading increasing, the steady dielectric permittivity @1.0k Hz presented an initial fast increase then slightly decrease trend, *e.g.* the CEC/PVCg elastomer with 9 wt% loading concentrations of CEC has a breakdown strength of 21.19 V/μm (**Supplementary Fig. 8b**). AC conductivity presented a continuous increase with the frequency increasing, its value evolved from 3.14×10^{-10} S/cm @ 1.0k Hz to 4.56×10^{-10} S/cm @ 1.0k Hz (**Supplementary Fig. 8c, 8d**).

Rotational rheometer (HAAKEMARS, USA) was used to measure the storage modulus G' (**Supplementary Fig. 9a**) and loss modulus G'' (**Supplementary Fig. 9b**) at room temperature. Each elastomer presented a typical elastic behavior, where its storage modulus (G') was much higher than its loss modulus (G''). With the continuous increase of CEC loading, G' exhibited a fast increase, while G'' exhibited a slight increase, thus the counted mechanical loss (**Supplementary Equation 6**) decreased. An extremely low mechanical loss (0.04 at 1 Hz) was presented.

Creep behaviors were observed by a cellphone of APPLE VII. Three PVCg films with dimensions of 50×4.0×1.1 mm^3 and three CEC/PVCg films with dimensions of 50×4.0×1.0

mm^3 were suspended by a constant load of 60 gf for 6 hours, the creep elongation was counted by the **Supplementary Equation 8** according to the captured images shown in **Supplementary Fig. 10**. The stress relaxation behaviors were tested by a universal tensile testing machine (UTM2202, China). Data of the stress attenuation were counted by the **Supplementary Equation 7**.

Supplementary Note 4: Actuation Behaviors

The flection amplitude of DEA from the diaphragm center was collected by a laser displacement sensor (Keyence LK, Japan) (**Supplementary Fig. 11**). According to the spherical crown model [7] shown in **Supplementary Fig. 12**, the areal strain was counted. Typical displacement output curves were shown in **Supplementary Fig. 13a**, and their counted areal strains were shown in **Supplementary Fig. 13b**. Results: with increasing CEC loading, the displacement output presented an initial increase following a decreasing trend, the maximum value appeared at the CEC loading of 9 wt%.

Supplementary Fig. 14 showed the relationship between the flection displacement and the driven frequency for the CEC/PVCg elastomer with 9 wt% loading concentrations of CEC. The CEC/PVCg elastomer actuator presented a characteristic Maxwell field driven DEA, where the flection amplitude decreases with the increase of frequency, because the electromechanical response of DEA always lags behind the change of electrical field. The total response time in one cycle varied in the range of 0.1-0.5 s.

To make a fair comparison, we made PDMS based DEA by using Dow Corning 186 and acrylic elastomer based DEA by using the commercial VHB 4910. Their electrodes were

adopted the CB/PDMS electrode, same to the PVCg based DEA. The actuation properties were measured under the same condition, where each pre-strain was fixed to 25 %. Results of the collected flection displacements and the counted area strains were shown in **Supplementary Fig. 15** and **Supplementary Table 2**. For PVCg actuator, its flection amplitude increased from 0.24 mm to 0.91 mm, and its areal strain increased from 0.81 % to 3.15 %, when the driven voltage increased from 5.45 V/μm to 9.09 V/μm (**Supplementary Fig. 15a**). For CEC/PVCg actuator, its flection amplitude increased from 0.43 mm to 3.23 mm, and its areal strain increased from 1.46 % to 12.22 %, when the driven voltage increased from 5.45 V/μm to 9.09 V/μm (**Supplementary Fig. 15b**). For PDMS actuator, its flection amplitude increased from 0.18 mm to 0.68 mm, and its areal strain increased from 0.65 % to 2.44 %, when the driven voltage increased from 5.85 V/μm to 9.07 V/μm (**Supplementary Fig. 15c**). For VHB actuator, its flection amplitude increased from 0.20 mm to 0.97 mm, and its areal strain increased from 0.72 % to 3.45 %, when the driven voltage increased from 12.50 V/μm to 22.50 V/μm (**Supplementary Fig. 15d**). It should be noted that, the common VHB actuator could not produce flection below the driven electrical field of 10.0 V/μm. In our actuation, the VHB actuator was triggered only when the driven electrical field was higher than 12.50V/μm.

Supplementary Fig. 16a-16e showed the relative displacement shift (RDS) for the four actuators. For varying actuation times, both the CEC/PVCg and PDMS actuators presented lower RDS values. By contrast, apparent displacement shifts were observed over time for PVCg and VHB 4910 actuators. RDS were calculated to quantify the viscoelastic effects. It was found that RDS values increased with time, *i.e.* number of actuation cycles, for VHB 4910 and PVCg actuators, while remaining almost constant for CEC/PVCg actuators. The relative shifts over

1000 cycles of CEC/PVCg actuators (7.78 % of RDS) represented 87 % and 94 % reductions as compared to PVCg (59.40 % of RDS) and VHB 4910 actuators (136.09 % of RDS). **Supplementary Fig. 16f** exhibited that the resultant CEC/PVCg actuator produced the maximum area strain below the driven electrical field of 20.0 V/μm.

Supplementary Note 5: Sensing Behaviors

DES was fixed by a self-made circle frame, a linear actuator pushed DES to take periodic out-of-plane strain, the capacitance signal from a single-chip microcomputer and displacement signal from the laser displacement sensor were synchronously collected (**Supplementary Fig. 17a**, **17b**) for comparisons. The collected capacitance signals were fitted to obtain the capacitance sensitivity (*S*) through the **Supplementary Equation 10**. For comparisons of sensors, we made PDMS based DES by using Dow Corning 186 and acrylic elastomer based DES by using the commercial VHB 4910. Their electrodes were adopted the CB/PDMS electrode, same to the PVCg based DES. Sensitivity tests were performed under same conditions to the PVCg based DES, shown in **Supplementary Fig. 18** and **Supplementary Table 2**. The CEC/PVCg sensor showed the higher sensitivity (*S*) than other three sensors under the same conditions. The sensitivity (*S*) of CEC/PVCg sensors were 1.8-fold and 3.1-fold higher than PDMS sensors in the displacement ranges of 0-7 mm and 7-14 mm. The sensitivity (*S*) of CEC/PVCg sensors were similar and 1.5-fold higher than VHB 4910 sensor in the displacement ranges of 0-7 mm and 7-14 mm.

Supplementary Fig. 19 showed relationship between the collected capacity and the driven frequency for the CEC/PVCg elastomer under a constant flection, where the CEC/PVCg

elastomer DES presented a fast sensing response time of 0.25-1 s.

Supplementary Fig. 20 reflected the high sensing stabilities of the CEC/PVCg and PDMS based DESs. The profiles of relative capacitance over time (**Supplementary Fig. 20a-20d**) indicated the stable performance for CEC/PVCg and PDMS sensors while apparent shift over time for PVCg and VHB 4910 sensors. The relative standard deviation (RSD) of relative capacitances over 1440 cycles was calculated to quantify the stability of their sensing performance. The results showed that our CEC/PVCg and PDMS sensors displayed much lower RSD values (5.75 % and 3.67 %), *i.e.* higher stability, than PVCg (9.84 %) and VHB 4910 (8.20 %) sensors.

Supplementary Table 3 compared with the commercial magnetic and strain sensors with our CEC/PVCg DES. For instance, the commercial strain gauge sensor has a very high sensitivity, but it was limited to a narrow range of distance *e.g.* 0-0.12 mm [8]. By contrast, Our CEC/PVCg sensors can achieve a high sensitivity of 3.08 pF/mm over a much wider range of deformation, *i.e.* 0-14 mm. In addition, the relative high sensitivity of existing sensors has been heavily relying on the sophisticated structural design and micro/nano-manufacturing technologies [9], such as micro-electromechanical systems (MEMS), resulting in the high complexity and high cost. By contrast, our CEC/PVCg sensor was fabricated by a simple mold-casting method and it costs ~ $ 2.00 per sensor.

Supplementary Equations

Supplementary Equation 1. Strain of DEA

$$\mathbf{S}_Z = -\frac{\varepsilon_0\,\varepsilon_r\,V^2}{Yd^2} = -\frac{\varepsilon_0\,\varepsilon_r\,\mathbf{E}^2}{Y} = -k\mathbf{E}^2 \quad (S1)$$

Where $\mathbf{S}_z$ is the thickness strain, ε_0 and ε_r are the permittivity of free space and the relative permittivity of the elastomer matrix, respectively, *Y* is the Young's modulus, **E** is the applied electrical field, *d* is the thickness of the matrix film, *k* is electromechanical coupling sensitivity (ε/Y), and *V* is driven voltage.

Supplementary Equation 2. Capacitance of DES

$$C = \frac{\varepsilon_0\varepsilon_r A}{d} \quad (S2)$$

Where *C* is the capacitance, ε_0 and ε_r are the permittivity of free space and the relative permittivity of elastomer matrix, respectively, *A* is the area, *d* is the thickness of matrix film.

Supplementary Equation 3. Degree of substitution

$$DS = \frac{(162\ \times \mathrm{N\%})}{(1400 - 53 \times \mathrm{N\%})} \quad (S3)$$

Where *DS* is degree of substitution, N % is N elemental content.

Supplementary Equation 4. Degree of crystallization

$$DC = \frac{I_{002} - I_{am}}{I_{002}} \times 100\% \quad (S4)$$

Where *DC* is the crystallinity of cellulose. Refer to literature [10], I_{002} is the diffraction intensity of the (002) crystal plane; I_{am} is the diffraction intensity of the amorphous region.

Supplementary Equation 5. CEC mass fraction

$$f_m = \frac{w_{CEC}}{(w_{PVC} + w_{CEC})} \times 100\% \quad (S5)$$

Where f_m is the mass fraction of CEC, w_{CEC}, w_{PVC} are the masses of CEC and PVCg, respectively.

Supplementary Equation 6. Mechanical loss

$$tan\,\delta = \frac{G''}{G'} \quad (S6)$$

Where (G') is storage modulus, G'' is loss modulus, tan δ represents mechanical loss.

Supplementary Equation 7. Stress attenuation

$$\boldsymbol{\sigma}_{\text{attenuation}} = \frac{\boldsymbol{\sigma}_0 - \boldsymbol{\sigma}}{\boldsymbol{\sigma}_0} \times 100\% \ \ (S7)$$

Where $\boldsymbol{\sigma}_{\text{attenuation}}$ is stress attenuation, $\boldsymbol{\sigma}_0$ and $\boldsymbol{\sigma}$ are the initial and terminal stresses, respectively.

Supplementary Equation 8. Creep elongation

$$\text{Creep elongation} = \frac{L - L_0}{L_0} \times 100\% \ \ (S8)$$

Where L_0 is the original length, L is the terminal length after creep.

Supplementary Equation 9. Relative displacement shift (RDS)

$$\text{Relative displacement shift (RDS)} = \frac{\mathbf{D} - \mathbf{D}_{creep}}{\mathbf{D}} \times 100\% \ \ (S9)$$

Where D is the original displacement of DEA in response to the electric field, $\mathbf{D}_{creep}$ is the creep displacement caused by the "baseline drift" in displacement-time curve [11].

Supplementary Equation 10. Capacitance sensitivity

$$S = \frac{\frac{\Delta C}{C_0}}{\Delta \mathbf{D}} \quad (S10-1)$$

$$\Delta C = C - C_0 \quad (S10-2)$$

$$\Delta \mathbf{D} = \mathbf{D} - \mathbf{D}_0 \quad (S10-3)$$

Where S is capacitance sensitivity, C_0 is the initial capacitance, C is the detected capacitance, ΔC and $\Delta \mathbf{D}$ are the capacitance difference and displacement difference, respectively.

Supplementary Equation 11. Relative standard deviations (RSD)

$$\bar{C} = \frac{C_1 + C_2 + \cdots C_n}{n} \qquad (S11 - 1)$$

$$SD = \sqrt{\frac{\sum_{i=1}^{n}(C_i - \bar{C})^2}{n}} \qquad (S11 - 2)$$

$$RSD = \frac{SD}{\bar{C}} \qquad (S11 - 3)$$

Where $C_1, C_2,\ C_i, C_n$ are the peak capacitances of *1, 2, i, n* cycles (n < 1440); $\bar{C}$ is the mean peak capacitance, SD and RSD is the standard deviation and relative standard deviation, respectively.

Supplementary Figures

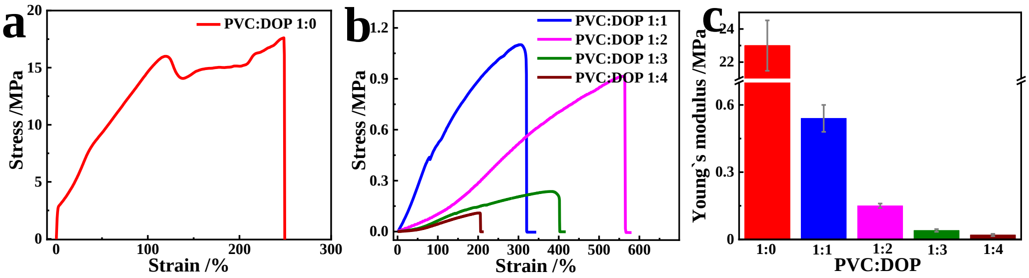

Supplementary Fig. 1. Mechanical property tests. Stress-strain curve of the self-casting PVC plastics (a); Typical stress-strain curves of PVCg elastomers with PVC: DOP mass ratios of 1:1, 1:2, 1:3, 1:4 (b); their counted Young's modulus (c). The error bars represent the standard deviations.

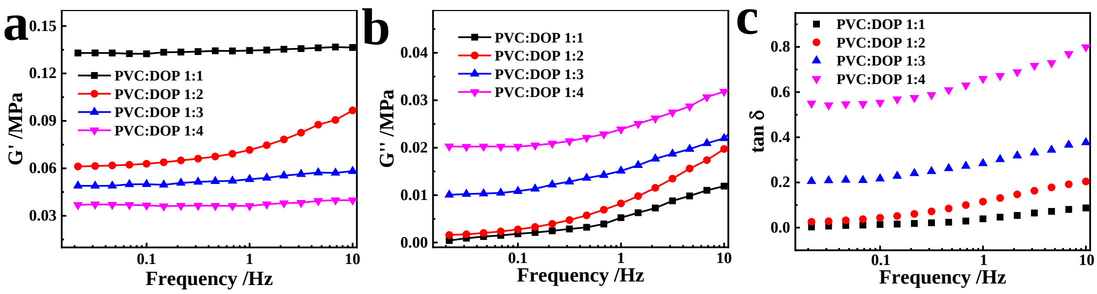

Supplementary Fig. 2. Dynamic viscoelasticity tests. Evolutions of storage moduli (G') (a), loss moduli (G'') (b), and the counted mechanical losses (tan δ = G''/G') (c) for PVCg elastomers with varying DOP concentrations under frequencies of 0.01-10 Hz.

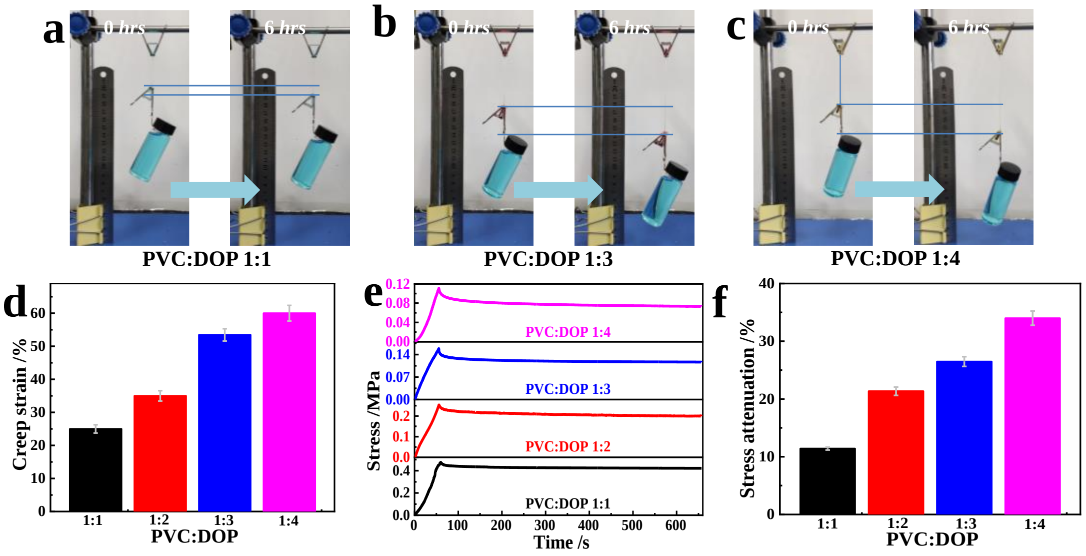

Supplementary Fig. 3. Static viscoelasticity tests. Static viscoelasticity of PVCg elastomers with varying DOP concentrations. Creep behaviors for PVCg elastomers with PVC: DOP mass ratios of (a) 1:1, (b) 1:3, (c) 1:4 under a constant load of 60 *g* for 6h (data of the elastomer of mass ratio of 1:2 was shown formerly). Quantifications of (d) creep strains, (e) stress relaxations, and (f) percentages of stress attenuation of PVCg elastomers with PVC: DOP mass ratios of 1:1, 1:2, 1:3, 1:4. The error bars represent the standard deviations.

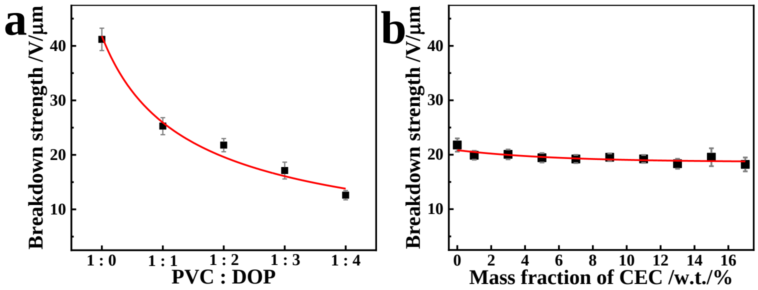

Supplementary Fig. 4. Breakdown strength tests. Evolution of breakdown strengths for PVCg elastomers with varying DOP concentrations (a). Evolution of breakdown strengths for CEC/PVCg elastomers with varying CEC concentrations (b). The error bars represent the standard deviations.

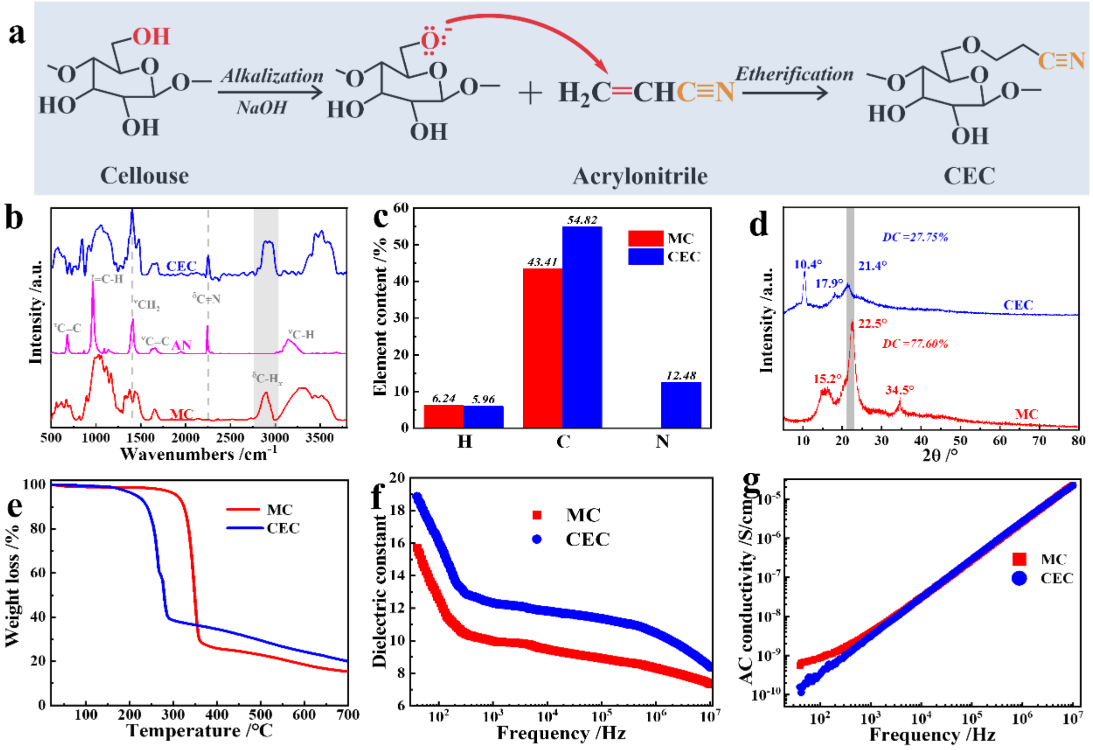

Supplementary Fig. 5. Synthesis and characterization of CEC. Synthesis routine (a); IR curves (b), elemental contents (c), XRD patterns (d), TG curves (e), dielectric constants (f), AC conductivities (g) for the pure MC and CEC.

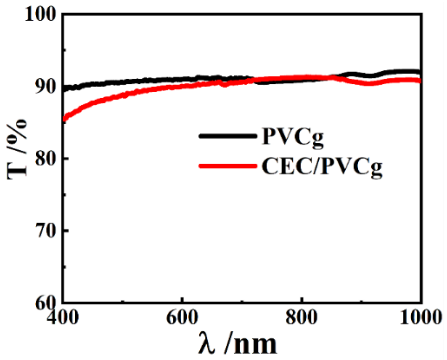

Supplementary Fig. 6. Light transmittances for PVCg and CEC/PVCg elastomers. For PVCg, the mass ratio of PVC: DOP was 1:2. For CEC/PVCg, the CEC mass loading was 9 wt%.

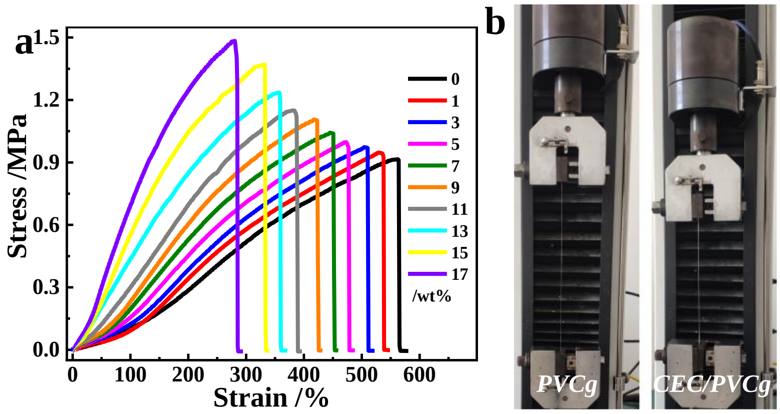

Supplementary Fig. 7. Mechanical property tests. Typical stress-strain curves of CEC/PVCg elastomers with varying CEC loadings (a); optical image of tensile tests for PVCg and CEC/PVCg elastomers (b). For PVCg, the mass ratio of PVC: DOP was 1:2. For CEC/PVCg, the CEC mass loadings varied from 0-17 wt%.

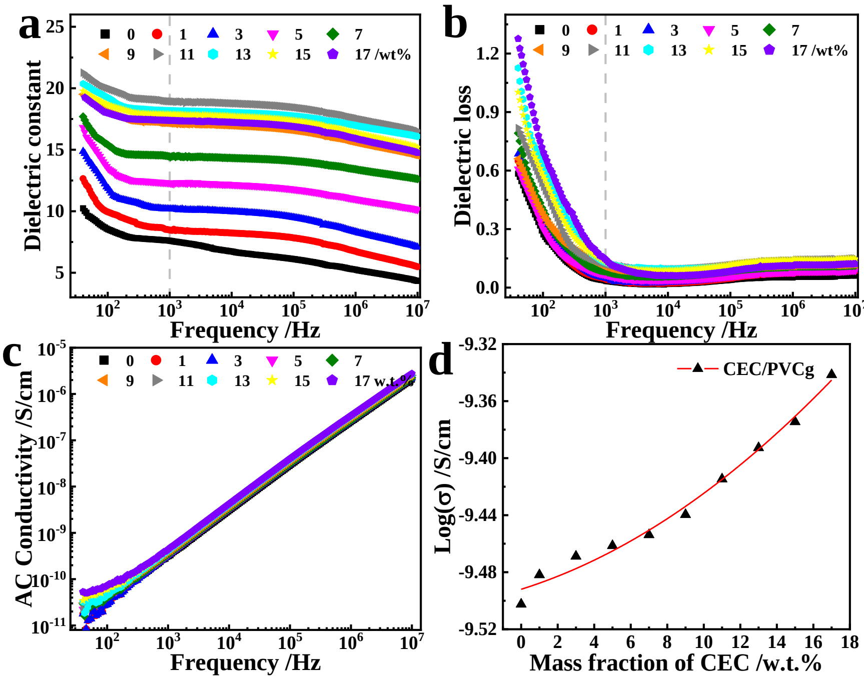

Supplementary Fig. 8. Dielectric property tests. Dielectric constants (a), dielectric losses (b) and AC conductivities (c) of CEC/PVCg elastomers with varying CEC loadings under 40-10^7 Hz frequencies. Evolution of AC conductivity at 1 kHz for CEC/PVCg elastomers (d). For CEC/PVCg, the CEC mass loadings varied from 0-17 wt%.

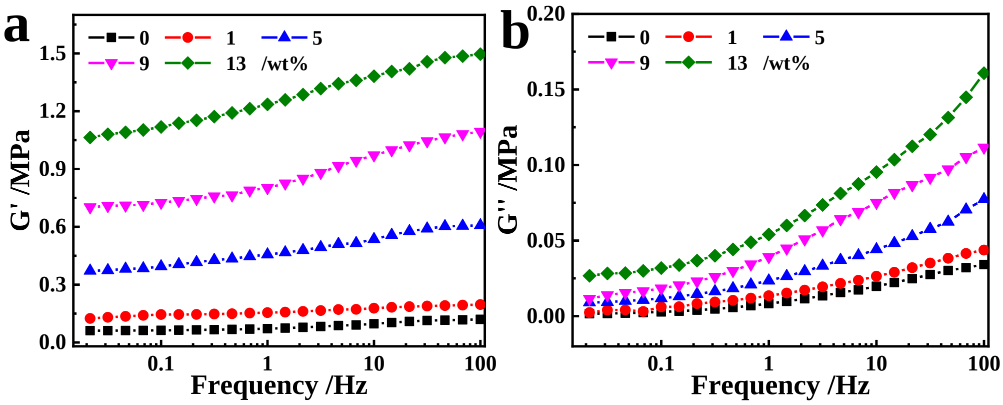

Supplementary Fig. 9. Dynamic viscoelasticity test. Evolutions of storage modulus (G') (a) and loss modulus (G") (b) for CEC/PVCg elastomers with CEC loadings of 0, 1, 5, 9, 13 wt% under frequencies of 0.01-100 Hz. For CEC/PVCg, the CEC mass loadings varied from 0-13 wt%.

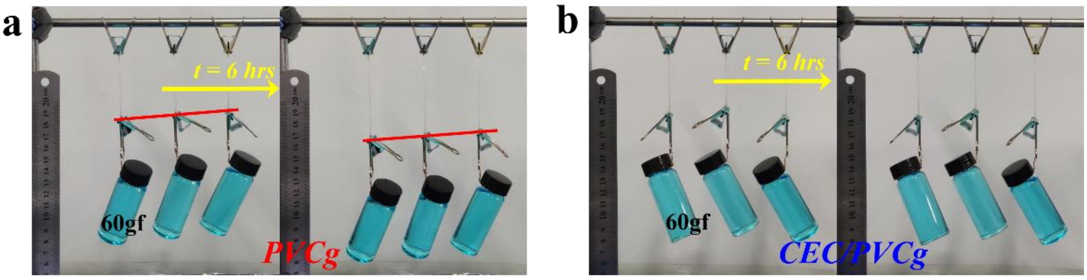

Supplementary Fig. 10. Creep evaluation tests. The creep elongations of PVCg (a) and CEC/PVCg (b) elastomers under a constant load of 60g for 6h. For PVCg, the mass ratio of PVC: DOP was 1:2. For CEC/PVCg, the CEC mass loading was 9 wt%.

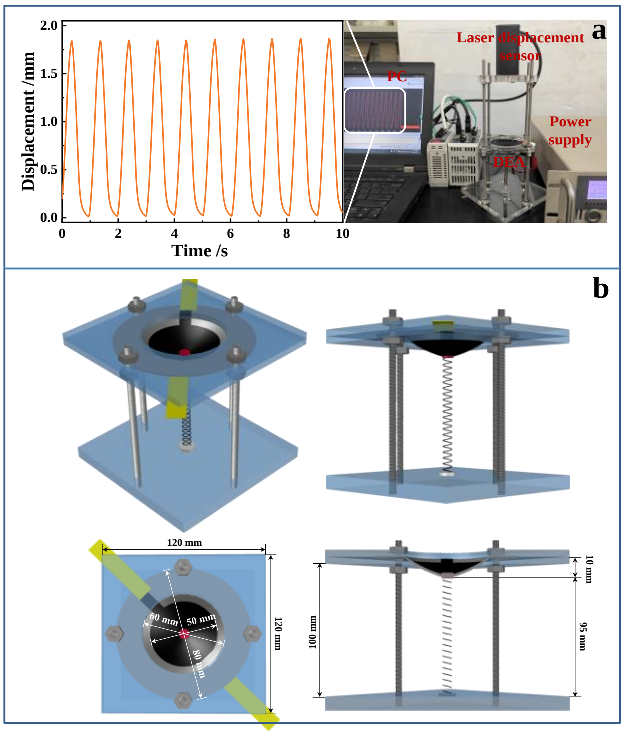

Supplementary Fig. 11. Testing platform for actuation. DEA's actuating platform (a); setup of DEA (b). The actuating platform is composed of a high-voltage power supply (ZMC-200, China), a laser displacement sensor (KEYENCY LK3001A, Japan). The high-voltage power supply can provide a pulsed electrical signal with an amplitude of 0-10 kV, a frequency of 0.1-10 Hz, and a duty ration of 25-75 %.

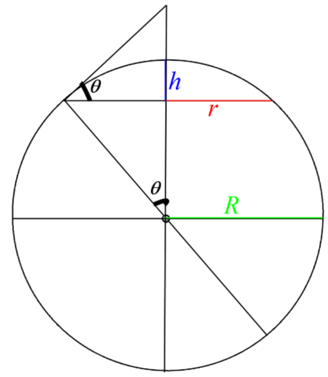

Supplementary Fig. 12. Schematic illumination for counting areal strain. The area strain of DEA can be calculated by the following equations.

$$S_A = \left(\frac{A}{A_0} - 1\right) \times 100\% = \left(\frac{h^2(1-\cos\theta_0)}{h_0^2(1-\cos\theta)} - 1\right) \times 100\%$$

$$A = 2\pi Rh$$

$$R = r/\sin\theta$$

$$h = R(1\text{-}\cos\theta)$$

Where S_A is the area strain; A is the area of spherical crown; A_0, r, and h are the area, radius, and height of sectional area, respectively; θ is the tangent angle; R is the ball radius.

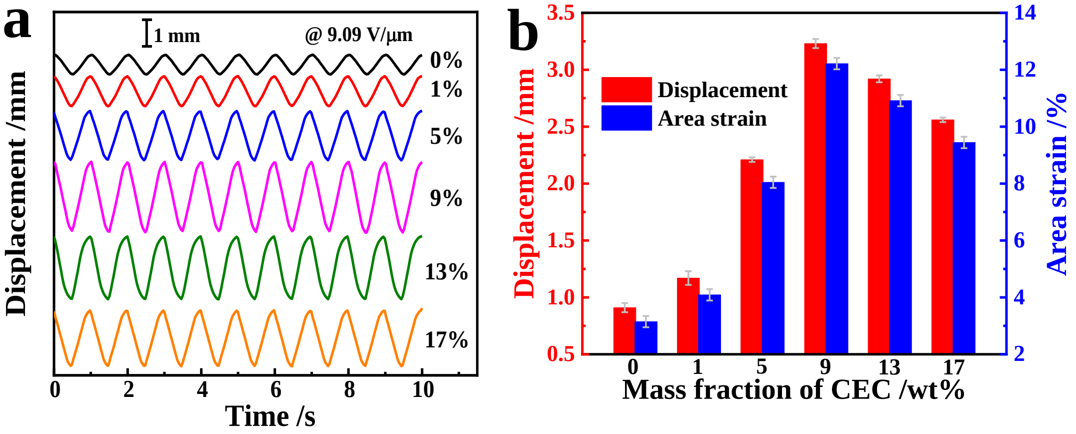

Supplementary Fig. 13. Actuation results. Typical displacement output curves of CEC/PVCg actuators with different CEC loadings under an electric field of 1 Hz and 9.09 V/μm (a); comparisons of the flection displacements and the counted area strains (b). The error bars represent the standard deviations.

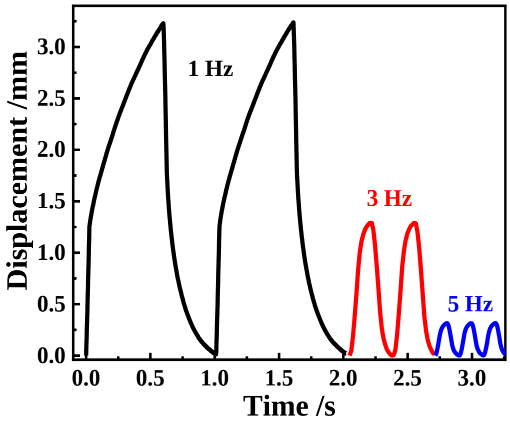

Supplementary Fig. 14. Response rate tests. Relationship between the flection displacement and the driven frequency for the CEC/PVCg elastomer with 9 wt% loading concentrations of

CEC under 9.09 V/μm electrical field.

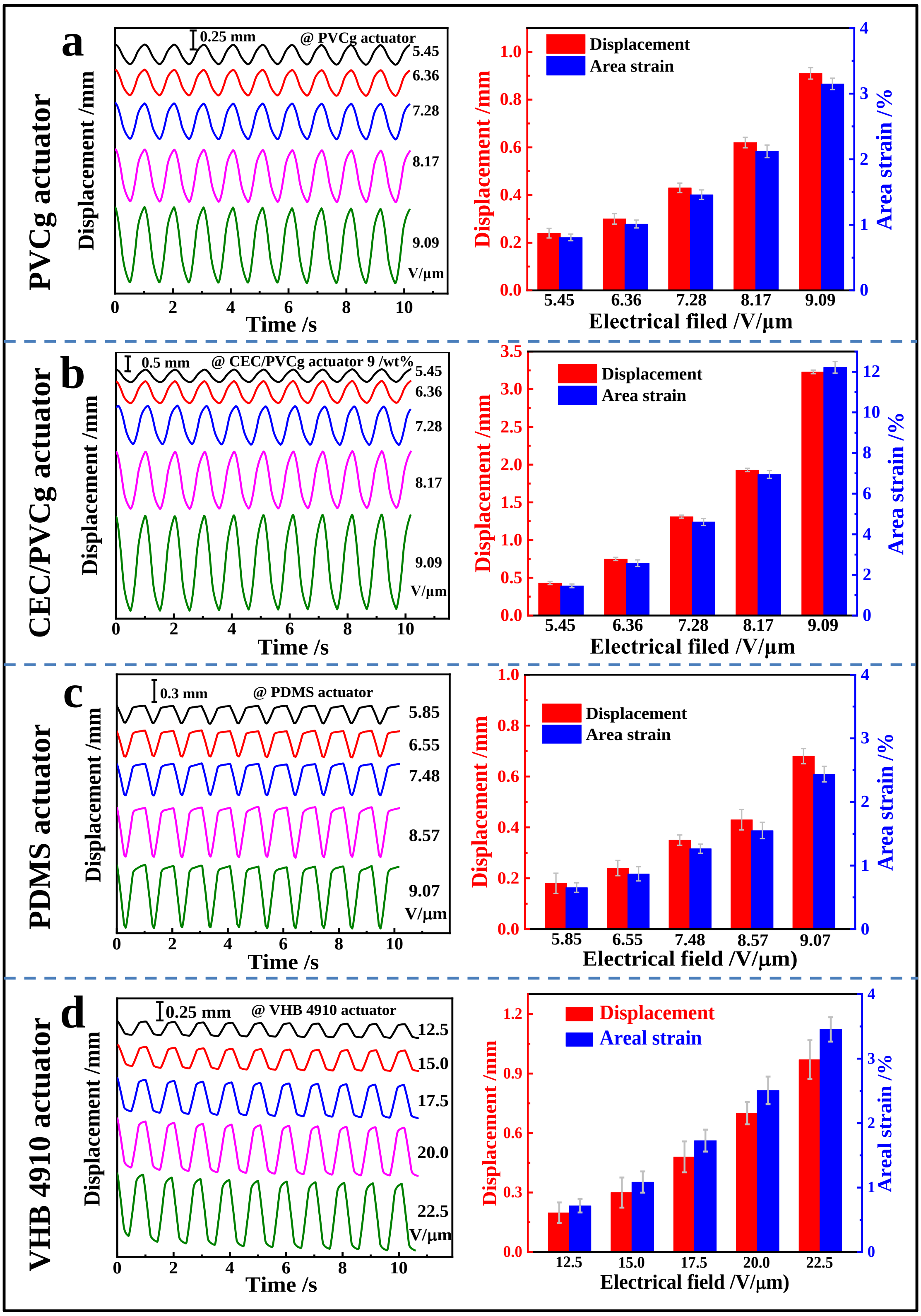

Supplementary Fig. 15. Actuation property comparisons. Typical displacement output curves, flection displacements and area strains of PVCg (a), CEC/PVCg (b), PDMS (c), VHB

(d) actuators with varying driven voltages. The error bars represent the standard deviations.

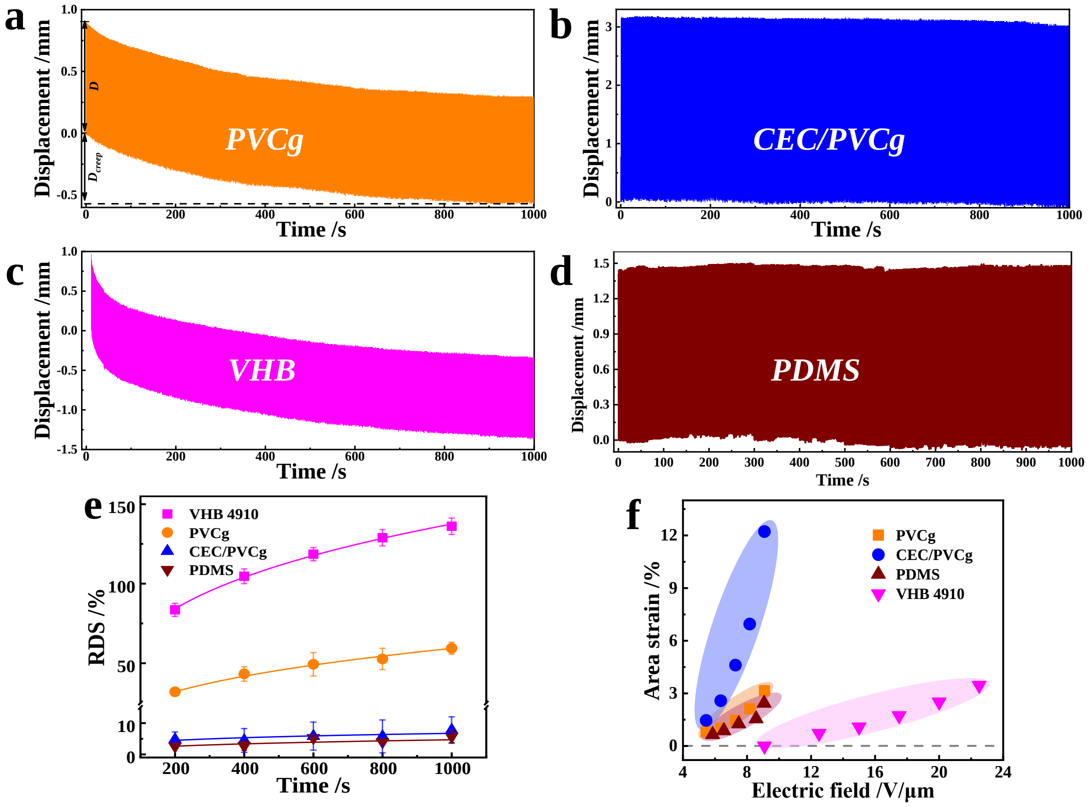

Supplementary Fig. 16. Actuation comparisons. Actuation stability and area strains of four types of actuators. Duration tests for the (a) PVCg, (b) CEC/PVCg, (c) VHB 4910, and (d) PDMS actuators over 1000 cycles of actuation, *i.e.* 1000 seconds. (e) Evolutions of relative displacement shift (RDS) of the four types of actuators. The actuation tests of PVCg, CEC/PVCg, and PDMS were performed under 9.09 V/μm electrical field and 1 Hz frequency; while the VHB actuator was triggered under 22.5 V/μm electrical field and 1 Hz frequency, *i.e.* $\text{RDS} = \frac{|\mathbf{D}-\mathbf{D}_{creep}|}{\mathbf{D}} \times 100\%$, where **D** is the amplitude of displacement and $\mathbf{D}_{\text{creep}}$ is the shift of the displacement as shown in (a). The error bars represent the standard deviations. (f) The mean values of area strains that were generated by four types of actuators as functions of driving electric fields.

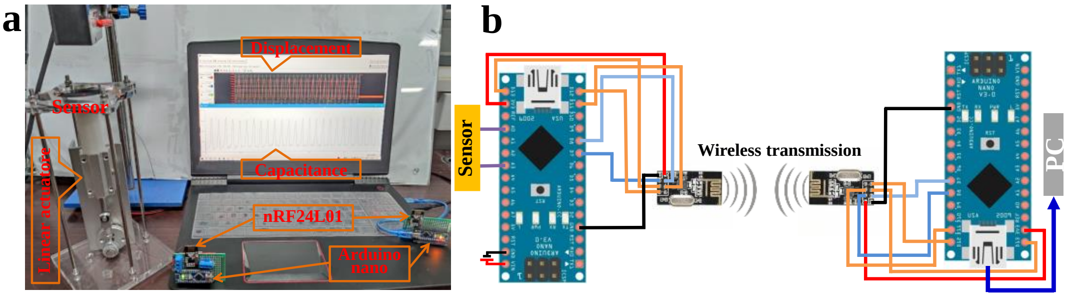

Supplementary Fig. 17. Testing platform for sensing. (a)Sensing behavior testing platform, which is made of a linear reciprocating actuator, (b) a commercial single-chip microcomputer (Arduino nano), and a wireless transmission module (nRF24L01).

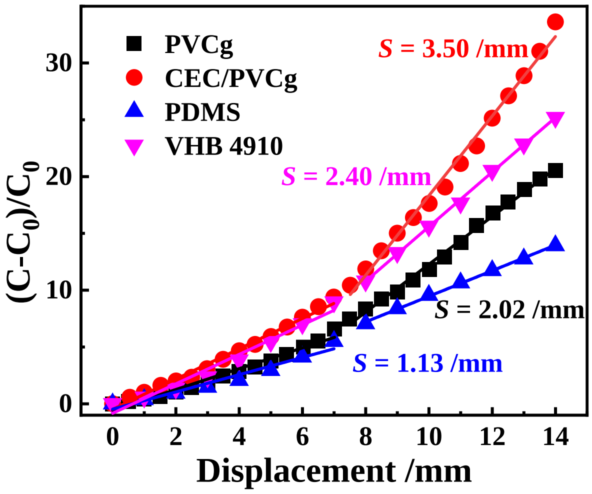

Supplementary Fig. 18. Sensitivity tests. The relative capacitance (C-C_0/C_0) that was generated by PVCg, CEC/PVCg, PDMS, and VHB sensors as a function of the flection displacement. The tangential slope of the curve was defined as the sensitivity (/mm) of the sensor.

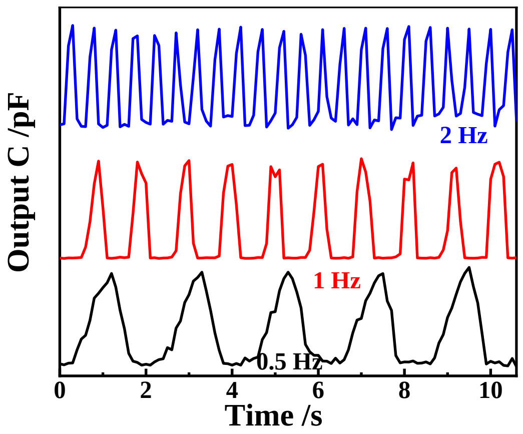

Supplementary Fig. 19. Response rate tests. Relationship between the collected capacity and the driven frequency for the CEC/PVCg elastomer under 14.0 mm flection displacement, which was generated from a commercial linear actuator. The CEC/PVCg sensors showed the fast response time, *e.g.* 1.0, 0.5, and 0.25 seconds under frequencies of 0.5-2.0 Hz.

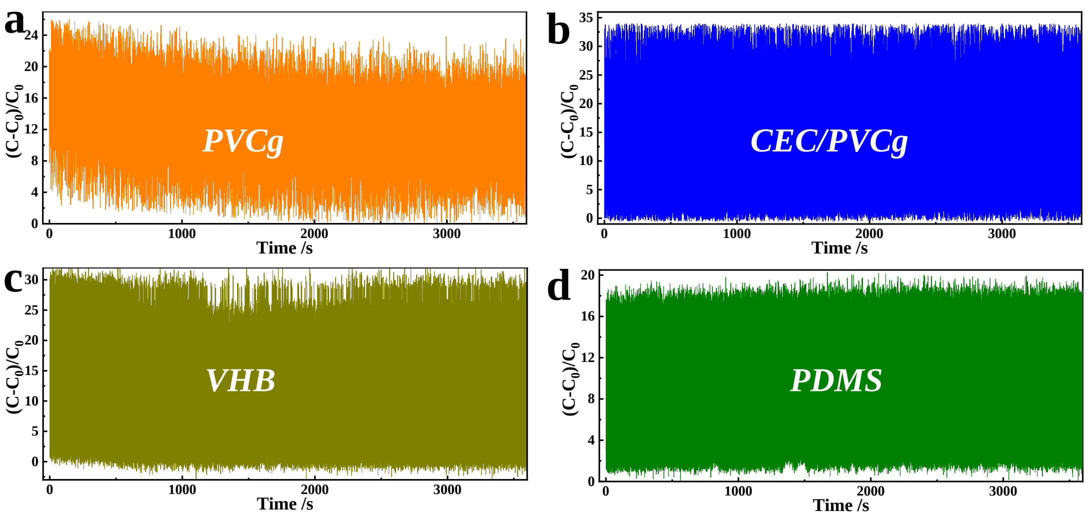

Supplementary Fig. 20. Stability tests. Duration tests within 1440 flection cycles (T = 2.5 s per cycle and 60 min in total) for the (a) PVCg, (b) CEC/PVCg, (c) VHB 4910, and (d) PDMS sensors. Comparison of the relative standard deviation (RSD = standard deviation of relative capacitance / mean of relative capacitance) values for the four types of sensors.

Supplementary Tables

Supplementary Table 1. Parameters of mechanical, dielectric properties of PVC matrix films.

Films with varying CEC loadings	Tensile strengths /MPa	Elongations at break /%	Moduli (Y) /MPa	Dielectric constants (ε) @1 kHz	Dielectric losses @ 1 kHz	Electromechanical sensitivities (ε/Y)
PVCg	0.91	563.80	0.15	7.59	0.033	50.60
CEC_1/PVCg	0.94	529.60	0.16	8.46	0.041	52.88
CEC_3/PVCg	0.97	504.15	0.18	10.18	0.048	56.56
CEC_5/PVCg	1.00	473.22	0.20	12.29	0.066	61.45
CEC_7/PVCg	1.04	445.82	0.22	14.40	0.083	65.45
CEC_9/PVCg	1.11	417.69	0.26	17.13	0.105	65.88
CEC_{11}/PVCg	1.15	382.69	0.30	18.93	0.121	63.10
CEC_{13}/PVCg	1.24	356.82	0.36	18.17	0.128	50.47
CEC_{15}/PVCg	1.37	331.56	0.42	17.86	0.132	42.52
CEC_{17}/PVCg	1.48	280.48	0.51	17.37	0.142	34.06

Supplementary Table 2. Physicochemical properties of VHB 4910, PDMS, PVCg, and CEC/PVCg elastomers, and their DEA/DES properties comparison.

		VHB	PDMS	PVCg	CEC/PVCg
Material properties	Dielectric constant@1kHz	4.40-4.70*	2.2-3.0**	7.59	17.13
	Dielectric loss@1kHz	0.020*	0.053**	0.033	0.105
	Dielectric loss tan *δ*@1kHz	0.0045*	0.0189**	0.0043	0.0061
	Young's modulus (*Y*) (MPa)	0.21*	0.70**	0.15	0.26
	Mechanical loss (tan *δ*) @1Hz	0.93*	0.088**	0.12	0.05
	Electromechanical sensitivity (*ε/Y*)	21.00*	4.00**	50.60	65.88
	Breakdown strength (V/μm)	28.4*	144**	21.79	19.53
Actuation properties	Pre-strain (%)	25	25	25	25
	Driving electric field (V/μm)	12.5-22.5	5.85-9.07	5.45-9.09	5.45-9.09
	Area strain (%)	0.72-3.45	0.65-2.44	0.81-3.15	1.46-12.22
	RDS@200s (%)	83.51	2.61	31.99	4.76
	RDS@1000s (%)	136.09	5.70	59.40	7.78
Sensing properties	Sensitivity (/mm)	2.40	1.13	2.02	3.50
	RSD of peaks (%)	8.20	3.67	9.84	5.75

* Material properties data of VHB came from the former report [12,13]; ** Material properties data of PDMS came from the former report [14].

Supplementary Table 3. Comparison of magnetic, strain, and capacitive sensors.

	Magnetic sensors*	Strain gage sensors**	Capacitive sensors*	CEC/PVCg Sensor
Stiffness	Rigid	Flexible	Flexible and stretchable	Flexible and stretchable
Range	100 nm-70 mm	0-0.12 mm	10 nm-10 μm	0-14 mm
Sensitivity	1.68 V/mm	Very high	0.038-5.3 pF/mm	3.08 pF/mm
Linearity (R^2)	0.9994	0.98-0.99	0.97-0.9975	0.988-0.992
Cost	Expensive	Expensive	Moderate	Cheap
Complexity	Complex	Complex	Complex	Simple

*Data of magnetic sensors and capacitive sensors referred to the literature [8]; **Data of strain gage transducer referred to the literature [15].

Supplementary References

1. Habashy, M. M., Abd-Elhady, A. M., Elsad, R. A. & Izzularab, M. A. Performance of PVC/SiO2 nanocomposites under thermal ageing. *Appl. Nanosci.* **11,** 2143-2151 (2021).
2. Wang, B., Kang, H. L., Yang, H. G., Xie, J. J. & Liu, R. G. Preparation and dielectric properties of porous cyanoethyl cellulose membranes. *Cellulose* **26,** 1261-1275 (2019).
3. Naduparambath, S. & Purushothaman, E. Sago seed shell: determination of the composition and isolation of microcrystalline cellulose (MCC). *Cellulose* **23,** 1803-1812 (2016).
4. Jia, C. et al. Barium titanate as a filler for improving the dielectric property of cyanoethyl cellulose/antimony tin oxide nanocomposite films. *Compos. Part A Appl. Sci. Manuf.* **86,** (2016) 1-8.
5. Kussmaul, B. et al. Enhancement of dielectric permittivity and electromechanical response in silicone elastomers: molecular grafting of organic dipoles to the macromolecular network. *Adv. Funct. Mater.* **21,** 4589-4594 (2011).
6. Hao, Y. et al. Significantly enhanced energy storage performance promoted by ultimate sized ferroelectric $BaTiO_3$ fillers in nanocomposite films. *Nano Energy* **31,** 49-56 (2017).
7. Guo, D. J., Han, Y. B., Ding, Y. H., Fang, S. M. & Tan, W. Prestrain-free electrostrictive film sandwiched by asymmetric electrodes for out-of-plane actuation. *Chem. Eng. J.* **352,** 876-885 (2018).
8. Stefanov, A. Z., Zivanov, L. D., Kisic, M. G. & Menicanin, A. B. Fully FFF-printed capacitive displacement sensor based on graphene/PLA composite and thermoplastic elastomer filaments. *IEEE Sens. J.* **22,** 10437-10445 (2022).

9. Ruth, S. R. A. et al. Rational design of capacitive pressure sensors based on pyramidal microstructures for specialized monitoring of biosignals. *Adv. Funct. Mater.* **30,** 1903100 (2020).

10. Segal, L., Creely, J. J., Martin, J. A. E. & Conrad, C. M. An empirical method for estimating the degree of crystallinity of native cellulose using the x-ray diffractometer. *Text. Res. J.* **29,** 786-794 (1959).

11. Zou, J., Gu, G. Y. & Zhu, L. M. Open-loop control of creep and vibration in dielectric elastomer actuators with phenomenological models. *IEEE-ASME T. Mech.* **22,** 51-58 (2017).

12. Yin, L. J. et al. Soft, tough, and fast polyacrylate dielectric elastomer for non-magnetic motor. *Nat. Commun.* **12,** 4517 (2021).

13. Liu, L. et al. Understanding reversible Maxwellian electroactuation in a 3M VHB dielectric elastomer with prestrain. *Polymer* **144,** 150-158 (2018).

14. Pan, C. F. et al. A liquid-metal–elastomer nanocomposite for stretchable dielectric materials. *Adv. Mater.* **31,** 1900663 (2019).

15. Araromi, O. A. et al. Ultra-sensitive and resilient compliant strain gauges for soft machines. *Nature* **587,** 219 (2020).
