# Supplementary material for: Polyvinyl chloride-based dielectric elastomer with high permittivity and low viscoelasticity for actuation and sensing
Source: Nat Commun. 2023 Mar 17;14:1483. doi: 10.1038/s41467-023-37178-5 (PMC10023783; doi:10.1038/s41467-023-37178-5)
Supplement: Supplementary file 3 — Description of Additional Supplementary Files [file 41467_2023_37178_MOESM3_ESM.pdf]

Description of Additional Supplementary Files

File Name: Supplementary Movie 1
Description: Out-of-plane actuation for the PVCg actuator under electrical field of 1.0 Hz and 9.09 V/µm.

File Name: Supplementary Movie 2
Description: Out-of-plane actuation for the CEC/PVCg actuator under electrical field of 1.0 Hz and 9.09 V/µm.

File Name: Supplementary Movie 3
Description: Force output for the PVCg actuator, where a 100 g weight was lifted under electrical field of 1.0 Hz and 9.09 V/µm.

File Name: Supplementary Movie 4
Description: Force output for the CEC/PVCg actuator, where a 300 g weight was lifted under electrical field of 1.0 Hz and 9.09 V/µm.

File Name: Supplementary Movie 5
Description: The CEC/PVCg sensor was triggered by a linear reciprocating actuator, and the resultant sensing signal was collected by a single-chip microcomputer.

File Name: Supplementary Movie 6
Description: The CEC/PVCg sensor was used to monitor the human knee' motion, whose sensing signal was collected online in real-time by a single-chip microcomputer and a wireless transmission module.
